# Chemical Composition and Antimicrobial Activity of Selected Essential Oils against *Staphylococcus* spp. Isolated from Human Semen

**DOI:** 10.3390/antibiotics9110765

**Published:** 2020-10-31

**Authors:** Miroslava Kačániová, Margarita Terentjeva, Jana Štefániková, Jana Žiarovská, Tatsiana Savitskaya, Dmitrij Grinshpan, Przemysław Łukasz Kowalczewski, Nenad Vukovic, Eva Tvrdá

**Affiliations:** 1 Department of Fruit Science, Viticulture and Enology, Faculty of Horticulture and Landscape Engineering, Slovak University of Agriculture, Tr. A. Hlinku 2, 94976 Nitra, Slovakia; 2 Department of Bioenergetics, Food Analysis and Microbiology, Institute of Food Technology and Nutrition, University of Rzeszow, Cwiklinskiej 1, 35-601 Rzeszow, Poland; 3 Institute of Food and Environmental Hygiene, Faculty of Veterinary Medicine, Latvia University of Life Sciences and Technologies, K. Helmaņa iela 8, LV-3004 Jelgava, Latvia; margarita.terentjeva@llu.lv; 4 AgroBioTech Research Centre, Slovak University of Agriculture, Tr. A. Hlinku 2, 94976 Nitra, Slovakia; jana.stefanikova@uniag.sk; 5 Department of Plant Genetics and Breeding, Faculty of Agrobiology and Food Resources, Slovak University of Agriculture, Tr. A. Hlinku 2, 949 76 Nitra, Slovakia; jana.ziarovska@uniag.sk; 6 Research Institute for Physical Chemical Problems, Belarusian State University, Leningradskaya str. 14, 220030 Minsk, Belarus; savitskayaTA@bsu.by (T.S.); Grinshpan@bsu.by (D.G.); 7 Department of Food Technology of Plant Origin, Poznań University of Life Sciences, 31 Wojska Polskiego St., 60-624 Poznań, Poland; przemyslaw.kowalczewski@up.poznan.pl; 8 Department of Chemistry, Faculty of Science, University of Kragujevac, P.O. Box 12, 34000 Kragujevac, Serbia; nvchem@yahoo.com; 9 Department of Animal Physiology, Faculty of Biotechnology and Food Sciences, Slovak University of Agriculture, Tr. A. Hlinku 2, 949 76 Nitra, Slovakia; eva.tvrda@uniag.sk

**Keywords:** *Staphylococcus* spp., human semen, essential oils, antimicrobial activity, antimicrobial resistance

## Abstract

*Staphylococcus* spp. is not only a commensal bacteria but also a major human pathogen that causes a wide range of clinical infections. Recent evidence suggests that *Staphylococcus* has the ability to colonize the reproductive system and to affect its structure and functions. The objective of this study was to determine the chemical properties and antibacterial effects of select essential oils (EOs): *Amyris balsamifera *L., *Boswellia carterii* Birdw., *Canarium luzonicum* (Blume) A. Gray, *Cinnamomum camphora* (L.) J. Presl., *Cinnamomum camphora* var. *linaloolifera* Y. Fuita, *Citrus x aurantium* L., *Gaultheria procumbens* L., *Litsea cubeba* (Lour.) Pers., *Melaleuca ericifolia* Smith., *Melaleuca leucadendra* L., *Pogostemon cablin* (Blanco) Benth., *Citrus limon* (L.) Osbeck, *Santalum album* L., and *Vetiveria zizanoides* (L.) Roberty against 50 *Staphylococcus* spp. cultures isolated from human semen, specifically *Staphylococcus aureus*, *S. capiti*, *S. epidermidis*, *S. haemoliticus*, and *S. hominis*. The disc diffusion and broth microdilution methods were used to assess the antimicrobial potential and to determine the minimum inhibitory concentration (MIC) of the selected EOs. The best anti-*Staphylococcus* activities were found with both methods for the essential oils of *C. luzonicum* (Blume) A. Gray, *A. balsamifera*, *C. camphora*, and *P. cabli*.

## 1. Introduction

As much as fifteen percent of infertility in males are a result of infections of the genitourinary tract [1]. Infections, both chronic and acute, lead to inflammation which compromises proper spermatogenic function [2,3,4]. This causes alterations in the sperm quality and quantity. Semen contamination occurs from microbiota present in the urinary tract or is transmitted via sexual intercourse [5].

*Staphylococcus* spp. has been frequently isolated from the reproductive system of men; furthermore, their ability to infect the male reproductive tract has been reported. *Staphylococcus* spp. may impair the secretory capacity of the epididymis, seminal vesicles, and prostate and may significantly affect sperm quality [6]. Essential oils (EOs) are a rich source of bioactive compounds, with some EOs exhibiting pronounced antimicrobial activity. Many plant parts, such as leaves, seeds, bark, resin, berries, flowers, roots, or fruits, contain EOs [7]. It has been shown that EOs of different plants and parts of the plant differ significantly in chemical composition and antimicrobial properties. Despite significant progress in the research of antimicrobial activity, extraction, and utilization of EOs, field studies on their application on opportunistic and pathogenic microbiota isolated from humans are needed [8]. Previous research suggests that the antimicrobial effect of EOs on human isolates could be used to prevent community- or hospital-acquired infections, which could become a suitable strategy to minimize the spread of antimicrobial resistance and to increase the efficiency of conservative treatment options [7,8,9].

The strongest antimicrobial activity of the *Juniperus communis* essential oil was found against *S. hominis* [10]. Salari et al. [11] used *Eucalyptus globulus* leaf extract to evaluate its activity on 56 isolates of *S. aureus*. The EOs extracted from all seven *Eucalyptus* spp. exhibited antibacterial activity against *S. aureus*. The best antimicrobial activity of *E. globulus* was found against *S. aureus* and *S. capiti*. In the meantime, *Cananga odorata* showed the best antimicrobial activity against *S. hominis* [10]. 

The objective of the present study was to investigate the chemical properties of selected essential oils and their antimicrobial effects against *Staphylococcus* spp. isolated from human semen.

## 2. Results and Discussion

### 2.1. Isolated Species of Staphylococci

**Figure 1:**
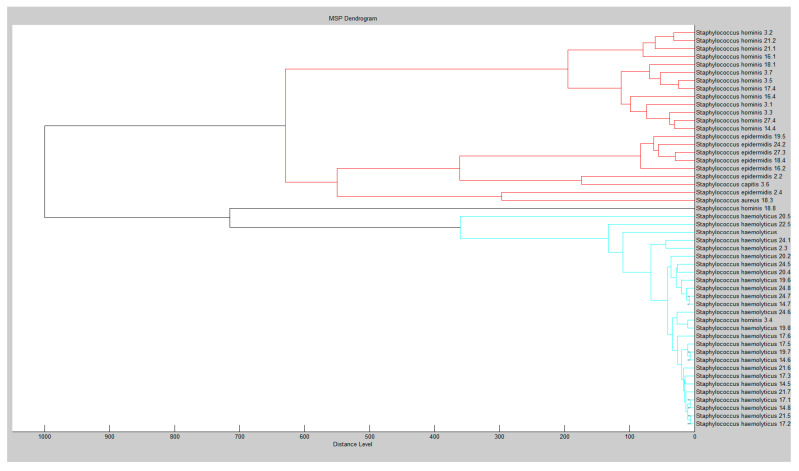
Dendogram of isolated *Staphylococcus* spp. from human semen constructed with a MALDI-TOF MS Biotyper.

In our study, 96 isolates were identified with mass spectrometry, with 50 isolates receiving a score higher than 2.00. The *Staphylococcus* spp. strains were *Staphylococcus aureus* (1 isolate), *S. capitis* (1 isolate), *S. epidermidis* (7 isolates), *S. haemoliticus* (26 isolates), and *S. hominis* (15 isolates) among the reliably identified isolates. The dendrogram of relatedness of mass spectra of *Staphylococcus* species is shown in Figure 1. 

Two main branches with multiple subbranches can be seen in the constructed dendrogram. The diversity of spectra of all *Staphylococcus haemolyticus* were obtained as more narrow while the spectra of *Staphylococcus epidermis* were most diverse in comparison to all other *Staphylococcus* spp. that were analysed. *Staphylococcus capitis* and *Staphylococcus aureus* were assigned to be similar to the *Staphylococcus epidermis* group according to their protein profiles. A third compact group was created for the mass spectra of *Staphylococcus hominis* with two isolates that were related to other branches.

Infertility has become a commonly observed clinical diagnosis with infections of the genital tract being frequently identified in patients who undergo assisted reproductive therapy [12]. 

Infections of the genital tract are caused by microorganisms transmitted from the urinary tract or sexually transmitted as a result of sexual activity. Changes in the morphology and motility of spermatozoa as well as a reduced sperm viability have been identified as a result of the infection [13]. Up to 34.4% of semen samples were found to be contaminated with microorganisms, predominantly with *Staphylococcus* spp., *Enterococcus*, and *Escherichia coli* [14].

### 2.2. Chemical Composition of Essential Oils

Different factors affecting the chemical composition of EOs. The most prominent endogenous factors are related to anatomical and physiological characteristics of the plants and to biosynthetic pathways of the volatiles, which might change depending on the plant tissue or season; however, it could also be influenced by DNA adaptation. On the other hand, exogenous factors might affect some of the genes responsible for volatiles formation, especially over a long period of time. Such changes may lead to ecotypes or chemotypes within the same plant species [15]. 

The chemical composition of *Amyris balsamifera* L. EO is shown in Table 1. The EO was obtained by steam distillation of crushed fresh wood. The presence of 15 chemical components with min 1% for each were identified. The compounds present in the highest amounts were valerianol (23.20%), guaiol (19.40%), and 10-epi-*γ*-eudesmol (14.80%). Different results were found in the study by Klouček et al. [16], where *α*-eudesmol (29.4%), *β*-eudesmol (10.4%), and valerianol (10.2%) were the main compounds of the amyris essential oil.

The chemical composition of *Boswelia carterii* Birdw. EO is given in Table 1. The EO was obtained by steam distillation of hand-collected resin. Nineteen chemical components with min 1% were identified. D-limonene (26.40%) and prehnitene (prehnitol, 8.65%) were the main compounds, which is in agreement with Camarda et al. [17].

The chemical composition of *Canarium luzonicum* (Blume) A. Gray EO is shown in Table 1. The EO was collected by steam distillation of resin. The presence of 12 chemical components with min 1% was found. The main compounds were D-limonene (36.40%) and elemol (16.70%), similar to the report of Villanueva et al. [18].

The chemical composition of *Cinnamomum camphora* (L.) J. Presl. EO is provided in Table 1. The EO was obtained by redistillation of wood and branches by steam, so-called white fraction, which does not contain safrole. Six chemical components with min 1% were found. The dominant constituents were 1,8-cineol (eucalyptol, 44.90%), D-limonene (25.90%), and *o*-cymene (11.70%). A previous study on the EO from fruits in the Guizhou province reported D-camphor (26.10%), 1,8-cineole (19.90%), linalool (9.20%), α-terpineol (7.20%), and limonene (5.30%) [19]. The main constituents in the sample from Jiangxi were D-camphor (42.80%), 1,8-cineole (24.80%), *α*-terpineol (8.70%), and *β*-pinene (5.80%) [20].

The chemical composition of *Cinnamomum caphora* var. *linaloolifera* Y. Fuita EO is presented in Table 1. The EO was acquired by steam distillation of leaves. The main compound was linalool (96.99%). Linalool was found to be the major constituent of *C. caphora* var. *linaloolifera* leaf oil (95.00%), with no other compounds present at a level of more than 1% [21].

The chemical composition of *Citrus x aurantium* L. EO is given in Table 1. The EO was obtained by distillation of fresh leaves. The presence of 11 chemical components with min 1% was recorded. The main compounds were linalyl acetate (63.40%) and α-terpineol (*p*-menth-1-en-8-ol, 8.84%), with linalool and linalyl acetate in leaves and limonene being found in previous studies [22,23].

The chemical composition of *Gaultheria procumbens* L. EO is presented in Table 1. The EO was acquired by distillation of freshly fermented fresh leaves. Methyl salicylate (98.00%) was the main compound which is in agreement with a previous report [24]

The chemical composition of *Litsea cubeba* (Lour.) Pers. EO is shown in Table 1. The EO was obtained by distillation of fruits. The presence of 11 chemical components with min 1% was found: (E)-citral ((F)-geranial and (E)-neral, 35.20%), (Z)-citral ((Z)-neral, 31.00%), and D-limonene (14.00%). Our results are in agreement with Thielmann and Muranyi [25], who stated that citral and limonene were the major components of *L. cubeba* EO extracted from fruits.

The chemical composition of *Melaleuca leucadendron* L. EO is given in Table 1. The EO was obtained by steam distillation of young shoots and leaves. The presence of 11 chemical components with min 1% was recorded. The main compounds were 1,8-cineol (eucalyptol, 49.20%) and α-terpineol (9.92%), which is line with previously reported 1,8-cineole (44.8–60.2%), α-terpineol (5.93–12.5%), D-limonene (4.45–8.85%), and β-caryophyllene (3.78–7.64%) [26].

The chemical composition of *Melaleuca ericifolia* Smith. EO is provided in Table 1. The EO was collected by steam distillation of branches. The presence of 13 chemical components with min 1% was observed. The main compounds were *β*-linalool (linalyl alcohol, 36.70%) and 1,8-cineol (eucalyptol, 23.10%). The EO from the leaves of *M. leucadendra* from Vietnam were found to be rich in *α*-eudesmol (17.6–21.2%) and guaiol (10.9–12.5%), and linalool was present in smaller concentrations (4.9–5.1%) [27]. Other studies indicated that 1,8-cineole was the major compound of *M. leucadendron* oil [28,29,30].

The chemical composition of *Pogostemon cabli* (Blanco) Benth. EO is given in Table 1. The EO was obtained by distillation of fermented leaves with steam, followed by maturation of the EO over time. Ten chemical components were present at min 1%, including patchouli alcohol (27.30%), *γ*-guajene (*α*-bulnesene, 18.20%), and *α*-guaien (18.10%). The major components of the oil were reported to be acetophenone (51.00%), *β*-pinene (5.30%), (E)-nerolidol (5.40%), and patchouli alcohol (14.00%) [31].

The chemical composition of *Citrus limon* (L.) Osbeck EO is displayed in Table 1. The EO was acquired by cold pressing fresh fruit. The presence of 8 chemical components that represented min 1% was recorded. D-limonene (67.10%) and *p*-mentha-1,4(8)-diene (iso-terpinene and *α*-terpinolene, 14.20%) were the main compounds while limonene (55.40%), neral (10.40%), trans-verbenol (6.43%), and decanal (3.25%) were found to be the main components among 43 identified compounds in the EO of this fruit in India [32].

The chemical composition of *Santalum album* L. EO is given in Table 1. The EO was obtained by steam distillation of crushed wood. Twelve chemical components were identified with a min 1%. The main compounds were α-santalol (59.00%), *α*-bergamotene (9.68%), and *β*-santalol (9.02%). Among those, α- and β-santalol, which accounted for 19.60% and 16.00%, respectively, were identified in India, and cis-*α*-santalol was recorded in the EOs from Sri Lanka [33,34].

The chemical coposition of *Vetiveria zizanoides* (L.) Roberty EO is presented in Table 1. The EO was obtained by steam distillation of sun-dried roots. The analysis indicated the presence of 28 chemical components at min 1%. The main compounds were β-vetivenene (7.42%) and khusenol (5.24%). David et al., 2009, analyzed oils extracted with carbon dioxide-expanded ethanol and found valerenol (18.50%), valerenal (10.20%), and *β*-cadinene (6.23%) to be the most common compounds out of a total of 23 molecules identified. Interestingly, 48 more components were found in oils processed with conventional hydrodistillation [35].

### 2.3. Antibacterial Effect of Antimicrobials

In this study, 50 isolates of *Staphylococcus* spp. acquired from human semen were tested for antimicrobial resistance (Table 2) against chloramphenicol, tetracycline, tigecycline, and tobramycin, and the results were interpreted according to the European Committee on Antimicrobial Susceptibility Testing (EUCAST) guidelines [36]. In total, 37 (74%) isolates were resistant while 13 (26%) isolates were sensitive to chloramphenicol. All tested isolates were sensitive to tetracycline and tigecycline. Resistance to tobramycin was identified in the case of 32 isolates, while 10 were sensitive and 8 were intermediately resistant to tobramycin.

Chloramphenicol is a broad spectrum antimicrobial which is active against gram-positive as well as gram-negative bacteria [37,38]. Because of chrolamphenicol toxicity and its application for life-treatening conditions, highly phenicol-resistant *S. aureus* strains of human origin have become a pressing area of scientific interest [39]. Resistance to tetracyclines is common as a result of their broad implementation in human and veterinary medicine. Furthermore, antimicrobial resistance to tetracycline has emerged in plants as well [40,41]. Resistance to tetracycline is encoded by genetic determinants and is fairly common in bacteria [42]. Tigecycline activity in vitro was observed against gram-positive and gram-negative microorganisms, such as *S. aureus*, *Enterococcus* spp., *S. pneumoniae*, *Haemophilus influenzae*, *Moraxella catarrhalis*, *Neisseria gonorrhoeae*, *N. peptostreptococci*, *Clostridium* spp., *Enterobacteriaceae*, and *Bacteroides* spp. [43,44]. It must be noted that differences in the antimicrobial resistance rates against gentamicin and tobramycin were found for *S. aureus* and *P. aeruginosa* across Europe [45].

### 2.4. Antimicrobial Assay

The antibacterial activities of 14 EOs against 50 *Staphylococcus* spp. isolates were determined with disc diffusion and broth dilution methods (Table 3, Table 4, Table 5 and Table 6). The antimicrobial properties of the assessed oils exhibited broad variations.

The best antimicrobial activity of *A. balsamifera* L. was found against *S. aureus* (16.50 ± 1.32 mm). *B. carterrii* Birdw. revealed the best antimicrobial effect against *S. epidermidis* (13.33 ± 1.15 mm). *C. luzonicum* (Blume) A. Gray showed the best antimicrobial activity against *S. capitis* (24.67 ± 0.58 mm), and *C. camphora* (L.) J. Presl. was found to be most effective against *S. hominis* (10.67 ± 0.58 mm). The best antimicrobial activity of *C. camphora* var. *linaloolifera* Y. Fuita was recorded against *S. aureus* (24.67 ± 0.58 mm), and *C. x aurantium* L. exhibited the highest antimicrobial properties against *S. epidermidis* (17.33 ± 0.58 mm). The EO of *G. procumbens* L. was most effective against *S. capitis* (8.33 ± 0.58 mm).

The best antimicrobial activity of *L. cubeba* (Lour.) Pers was found against *S. capitis* (25.33 ± 0.58 mm). *M. leucadendron* L. showed the best antimicrobial effect against *S. hominis* (7.67 ± 0.58 and 7.67 ± 1.15 mm, respectively). *M. ericifolia* Smith. was highly effective against *S. hominis* (15.33 ± 0.58 mm), while *P. cabli* (Blanco) Benth. exhibited the highest antimicrobial potential against *S. haemoliticus* and *S. hominis* (12.67 mm). The best antimicrobial activity of *C. limon* (L.) Osbeck was found against *S. capitis* (12.67 ± 1.15 mm), and *S. album* L. was highly efficient against *S. hominis* (8.67 ± 0.58 mm). The most effective antimicrobial activity of *V. zizanoides* (L.) Roberty EO was recorded against *S. capitis* and *S. hominis* (12.67 mm).

For the analysed EOs, significant differences in their activity were observed against *Staphylococcus* spp. (Table 7). The most pronounced activity was recorded for *C.luzonicum* (Blume) A. Gray, *A. Balsamifera* L., *C. camphora* var*. linaloolifera*, and *P. cabli* (Blanco) Benth. EOs.

**Figure 2:**
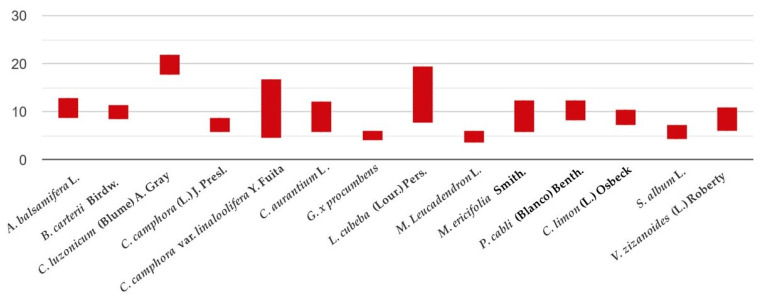
Mean (mm) and standard deviation for analysed essential oils in their activity against *Staphylococcus* spp.

No significant differences were found against *A. balsamifera* L. vs. *P. cabli* (Blanco) Benth.; *G. procumbens* L. vs. *M. leucadendron* L.; *B. carterii* Birdw. vs. *P. cabli* (Blanco) Benth.; *M. ericifolia* Smith. vs. *V. zizanoides* (L.) Roberty; *B. carterii* Birdw. vs. *C. camphora* var. *linaloolifera* Y. Fuita; *C. x aurantium* L. vs. *V. zizanoides* (L.) Roberty; *C. limon* (L.) Osbeck vs. *V. zizanoides* (L.) Roberty; *M. ericifolia* Smith. vs. *C. limon* (L.) Osbeck; *C. camphora* var. *linaloolifera* Y. Fuita vs. *P. cabli* (Blanco) Benth.; *C. x aurantium* L. vs. *C. limon* (L.) Osbeck; *A. balsamifera* L. vs. *C. camphora* var. *linaloolifera* Y. Fuita; and *C. x aurantium* L. vs. *M. ericifolia* Smith (Figure 2).

In this study, the EO of *A. balsamifera* L. showed the best antimicrobial activity with the disc diffusion test against *S. aureus* with an inhibition zone of 16.50 mm. Minimum inhibitory concentration (MIC) values obtained with the broth microdilution method were 1.59 µL/mL against *S. aureus*, *S. capitis*, one strain of *S. epidermidis*, 10 strains of *S. haemoliticus*, and three strains of *S. hominis*. *A. balsamifera* was reported to possess antimicrobial activity against gram-positive and gram-negative bacteria, including *Staphylococcus aureus*, *Salmonella paratyphi*, *Escherichia coli*, *Klebsiella pneumoniae*, and microscopic fungi [46].

*B. carterii* Birdw. EO was found to be the most effective against one strain of *S. epidermidis* (13.33 mm) tested with the disc diffusion method. With the microdilution method, MIC = 1.59 µL/mL was found against *S. aureus*, all strains of *S. epidermidis*, three strains of *S. haemoliticus*, and three strains of *S. hominis*. The antimicrobial activity of EOs of *B. carteri*, *B. neglecta*, *B. sacra*, *B. thurifera*, and *B. frereana* varied from moderate to poor against *S. aureus* (ATCC 12600) [47].

The EO of *C. luzonicum* (Blume) A. Gray exhibited the best antimicrobial activity against *S. capitis* (24.67 mm) with the disc diffusion method. Using the broth microdilitution method, MIC = 0.39 µL/mL was recorded against *S. aureus*, one strain of *S. epidermidis*, and one strain of *S. hominis*. *C. luzonicum* was reported to show antifungal activity without expressing toxicity or other negative side effects [48].

*C. camphora* (L.) J. Presl. EO revealed the best antimicrobial activity against *S. homins* with an inhibition zone of 10.67 mm with the disc diffusion test and MIC = 3.12 µL/mL against two strains of *S. haemoliticus*. *C. camphora* var. *linaloolifera* Y. Fuita showed the best antimicrobial activity against *S. aureus*, with an inhibition zone of 24.67 mm with the disc diffusion method and MIC = 0.39 µL/mL against *S. aureus.* The EO of *C. camphora* was found to possess antifungal activity against *A. niger* (MIC = 20 μg/mL) and exhibited an inhibitory effect against *B. cereus* and *S. aureus* [49]. Previously identified antimicrobial properties of the EOs of *C. camphora* were in agreement with our results [50,51,52,53,54].

The EO of *C. x aurantium* was the most active against one strain of *S. epidermidis* with the disc diffusion method (inhibition zone of 17.33 mm). With the broth microdilution method, MIC = 3.12 µL/mL was found against *S. aureus* and all strains of *S. epidermidis*. *C. aurantium* was found to inhibit *B. subtilis* and *P. crustosum* [55]. A study on the antimicrobial activity of the *C. aurantium* EO against pathogenic bacteria (*Staphylococcus aureus*, *Salmonella* sp., *Pseudomonas aeruginosa*, *Bacillus subtilis*, and *Escherichia coli)* revealed that gram-positive bacteria were more susceptible than gram-negative bacteria [56].

*G. procumbens* L. EO exhibited the strongest antimicrobial activity against one strain of *S. aureus* with the disc diffusion test (7.33 mm). An MIC value of 12.50 µL/mL was found for *S. aureus*, *S. capitis*, and one strain of *S. haemoliticus*, determined with the broth microdilution method. Hammer et al. [57] reported a higher activity of *G. procumbens* EO against reference strains of gram-negative bacteria (*Acinetobacter baumanii*, *Aeromonas sobria*, *Escherichia coli*, *Klebsiella pneumoniae*, *Salmonella typhi*, and *Serratia marcescens*) observed in comparison to gram-positive microorganisms (*Staphylococcus aureus* and *Enterococcus faecalis*). A higher resistance of gram-positive bacteria against *G. procumbens* EO was shown by Nikolic et al. [24], who studied the bacteriostatic and bactericidal activity of the oil against microbial isolates.

*L. cubeba* (Lour.) Pers. EO exhibited the best antimicrobial activity against *S. capitis* with the disc diffusion test (25.33 mm) and an MIC of 0.39 µL/mL against *S. aureus* and *S. capitis* with the broth microdilitution test. The antibacterial activity of *L. cubeba* EO against food-borne pathogens has been reported as well [58,59,60]. A notably high antimicrobial activity was found against methicyllin-resistant *Staphylococcus aureus* (MRSA) [61,62].

The EOs of *M. ericifolia* Smith showed the strongest antimicrobial activity against *S. aureus* with respect to *S. hominis*. *Melaleuca* EOs have been reported to possess antibacterial activity against common food-borne pathogens [63] and were suggested for the eradication of MRSA in hospitals [64]. Even a concentration of 5% *M. alternifolia* was active against pathogenic bacteria of skin, and a potential application of *M. alternifolia* oil for wound treatment was suggested as well [65,66,67]. Furthermore, antimicrobial, antifungal, antiviral, and antioxidant properties were described in *M. ericifolia* [26]. Leaf extracts acquired from this plant exhibited antimicrobial activity against gram-positive and gram-negative bacteria, including *S. aureus* [68].

The EO of *Pogestemon cabli* was the most effective against two strains of *S. haemoliticus* and *S. homins* (inhibition zone of 12.67 mm) using the disc diffusion method. The recorded MIC values against two strains of *S. aureus*, *S. capitis*, all strains of *S. haemoliticus*, and all but two strains of *S. hominis* were 3.12 µL/mL. The EO from *P. cabli* was found to be more active against gram-positive than gram-negative bacteria, with the largest inhibition zone (35 mm with 20 µL of oil) and the lowest MIC (250 µg/mL) and minimum bactericidal concentration (MBC) (750 µg/mL) found against *Bacillus cereus*. A moderate antifungal activity was recorded against *Candida albicans* in comparison to *Saccharomyces cerevisiae* (16- vs. 14-mm zone diameters with 20 µL of oil). The lowest MIC and minimal fungicidal concentration(MFC) (both were 750 µg/mL) were found for *Candida albicans* [69].

The EO of *C. limon* (L.) Osbeck was found to be the most effective against one strain of *S. capitis*, with an inhibition zone of 12.67 mm with the disc diffusion test. The broth microdilution method showed MICs of 3.12 µL/mL against *S. aureus, S. capitis*, as well as several strains of *S. haemoliticus* and *S. hominis*. The antimicrobial activity of EOs from *C. limon* was recorded against *S. aureus*, *E. coli*, and *B. subtilis* [70], with inhibitory effects against gram-positive bacteria [71]. Hydro-distillated EOs from *C. limon* were reported to be more active due to a high content of limonene [72].

The EOs of *S. album* L. exhibited the highest antimicrobial activity against one strain of *S. hominis* (8.67 mm). An MIC of 6.25 µL/mL was detected against *S. capitis* and all strains of *S. hominis*. A previously reported MIC for *S. album* ranged between 0.078 and 5 μg/mL [73], and an antimicrobial activity against *Staphylococcus aureus* and *Klebsiella pneumoniae* was described as well [74].

The EO of *V. zizanioides* (L.) Roberty showed the highest activity against *S. capitis* and one strain of *S. homins* with an inhibition zone of 12.67 mm using the disc diffusion test. With the broth microdilution tests, the MIC was 3.12 µL/mL against *S. aureus, S. capitis*, and all strains *S. hominis*. Gupta et al. [75] found a higher antimicrobial activity of the EO against gram-positive in comparison to gram-negative bacteria. Antifungal and antimicrobial activity against *Candida albicans* as well as wildtype and drug-resistant strains of *M. smegmatis* and drug-resistant strains of *E. coli* have been previously reported [76].

## 3. Materials and Methods

### 3.1. Essential Oil Samples

The following essential oils were used in the present study (Table 8): *Amyris balsamifera* L., *Boswellia carterii* Birdw., *Canarium luzonicum* (Blume) A. Gray, *Cinnamomum camphora* (L.) J. Presl., *Cinnamomum camphora* var. *linaloolifera* Y. Fuita, *Citrus x aurantium* L., *Gaultheria procumbens* L., *Litsea cubeba* (Lour.) Pers., *Melaleuca ericifolia* Smith., *Melaleuca leucadendra* L., *Pogostemon cablin* (Blanco) Benth., *Citrus limon* (L.) Osbeck, *Santalum album* L., and *Vetiveria zizanoides* (L.) Roberty. All EOs were produced in Slovakia (Hanus a.s., Nitra) and used in original packaging. All tested oils were stored in the dark at 4 °C.

### 3.2. Chemical Composition of EOs

Gas chromatographic-mass spectrometric analysis (GC Agilent 7890B and MS Agilent 5977A, Agilent Technologies Inc., Santa Clara, CA, USA) of the EOs was performed as described by Kačániová et al. [77] with a slightly modified version. Prior to the analysis, EO samples were diluted in hexane (HPLC ≥ 97%, Sigma Aldrich GmbH, Darmstad, Germany) to a concentration of 10 µL/mL. One microliter of diluted sample was injected into the inlet (250 °C) operated in split mode 1:10. The separation was achieved using a HP-5ms capillary column (30 m × 0.25 mm × 0.25 µm film; Agilent Technologies). The oven temperature program was set to 50 °C for the first 5 min and subsequently increased to 240 °C at the rate of 3 °C/min, where it was kept constant for 2 min. Helium was used as a carrier gas at constant flow (1.2 mL/min). The mass detector parameters were as follows: ionization energy of the filament—70 eV, transfer line temperature—250 °C, MS source temperature—230 °C, and quadrupole temperature—150 °C. The mass spectrometer was programmed under electron impact (EI) in a full scan mode at *m*/*z* 40–350 with a scanning rate of 2.4 scans/s. The identification of compounds was carried out by comparing mass spectra (over 80% match) with a commercial database NIST^®^ 2017 and the Wiley library for retention times of reference standards (D-limonene, *β*-myrcene, and *γ*-terpinene; Sigma-Aldrich GmbH) to compare data on occurrence in EOs with the literature. The relative content of the identified compounds was calculated by dividing the individual peak area by the total area of all peaks. Peaks under 1% were not counted. Each sample was measured in triplicate.

### 3.3. Microorganisms

Semen samples were obtained from 27 males following 2 days of sexual abstinence. The specimens were taken by masturbation into a sterile wide mouth container. The samples were liquefied at 37 °C for 30 min. All experiments were performed within 1 h after sampling. Only ejaculates showing normal semen parameters (concentration > 20 × 10^6^/mL, motility > 40%, viability > 40%, and morphology > 4%) and free from leukocytes were used. The experiments were approved by the Ethic Committee at the Specialized Hospital Sv. Svodar Zobor, protocol no. 030809/2015. Tryptone Soya agar (TSA, Merck, Darmstadt, Germany) and Blood agar (BA, Merck, Darmstadt, Germany) were inoculated with the semen samples, and after incubation (24 h, 37 °C), individual colonies were selected for further confirmation with MALDI-TOF MS Biotyper (Brucker Daltonics, Bremen, Germany) [78]. The isolates were maintained in Mueller Hinton Agar (MHA, Merck, Darmstadt, Germany) and cultured 24 h before the experiment to reach a concentration of 10^5^ cfu/mL.

### 3.4. Antimicrobial Susceptibility Testing

The antimicrobial susceptibility test was performed with the disc diffusion method against (10 mcg) chloramphenicol, tetracycline, tigecycline, and tobramycin. The discs were obtained from Oxoid (Basingstoke, UK). The results were interpreted according to EUCAST [36].

### 3.5. Disc Diffusion Method

A suspension of the tested culture (0.1 mL of 10^5^ cells/mL) was spread onto Mueller Hinton Agar (MHA, Oxoid, Basingstoke, UK). Filter paper discs (6 mm) were impregnated with 15 µL of the EO and placed on the inoculated plates. The agars were incubated at 4 °C for 2 h and subsequently placed into an incubator at 37 °C for 24 h. The diameters of the inhibition zones were measured in mm. All the tests were performed in triplicate [79]. The results were evaluated as follows (disk diameter included): ≥15 mm was strongly inhibitory, <15–10 mm was moderately/mildly inhibitory, and <10 mm was not inhibitory [78,79,80,81,82].

### 3.6. Determination of Minimum Inhibitory Concentration

The broth microdilution assay was used for determination of the minimal inhibition concentration (MIC) according to the Clinical and Laboratory Standards Institute [83]. All tests were performed in Mueller Hinton Broth (MHB, Oxoid, Basingstoke, UK). The bacterial strains were cultured overnight at 37 °C in MHA. The tested strains were suspended in MHB to give a final density of 10^6^ cfu/mL confirmed by viable counts. The EO solution was prepared in dimethyl sulphoxide (DMSO, Penta, Prague, Czech Republic). An amount of 50 μL of MHB was added to each 96-well micro-titer plate, and 100 μL of MHB was added to the 10th well for sterility control. For the growth control, MHB with 5% DMSO was added to the 9th well. Fifty microliters of EOs initially dissolved in 5% DMSO were added into the first well. A serial 2-fold dilution was performed by transferring 50 μL of the suspension to the subsequent wells up to the 8th well; bacterial inoculum of 0.5 McFarland was diluted in the ratio of 1:100 and added into the 1st–8th wells in order to acheive the final concentration of 5 × 10^5^ cfu/mL. Bacterial cell viability and MIC values were determined by observing the turbidity. The lowest concentrations of the EOs with clear suspension were considered as the MIC values. The test was performed in triplicate alongside cefoxitin (30 mcg), used as a positive control.

### 3.7. Statistical Analysis

The basic variation (disc diffusion method) in statistical values from obtained data were calculated with Statgraphic, Tukey HSD test. Mean, standard deviation, minimum, maximum, coefficient of variation, and frequency of size of inhibition zones were calculated for the antimicrobial activity of essential oils.

## 4. Conclusions

In this study, 50 different strains of *Staphylococcus* spp. isolated from human semen were tested for susceptibility against 14 different essential oils alongside determination of their chemical composition. The antimicrobial resistance of the tested isolates was evaluated as well. The antimicrobial resistance of *Staphylococcus* spp. against chloramphenicol and tobramycin was found, while all isolates were sensitive to tetracycline and tigecycline. *C. luzonicum* (Blume) A. Gray exhibited a strong inhibitory effect; *A. balsamifera* L., *C. camphora* var. *linaloolifera* Y. Fuita, *L. cubeba* (Lour.) Pers., and *P. cabli* (Blanco) Benth. possessed a moderately inhibitory effect; and *B. carterii* Birdw., *C. camphora* (L.) J. Presl., *C. aurantium* L., *G. procumbens* L., *M. leucadendron* L., *M. ericifolia* Smith., *C. limon* (L.) Osbeck, *S. album* L., and *V. zizanoides* (L.) Roberty revealed no inhibitory activity on *Staphylococcus* spp. isolated from human ejaculates. As such, we may suggest the use of the selected essential oils against *Staphylococcus* spp. contamination of human semen samples.

## Figures and Tables

**Table 1:** Chemical composition of essential oil (%) *.

Essential Oil	Components	RT (min.)	Percentage of Components (n = 3)
*Amyris balsamifera* L.	Amorpha-4,11-diene	24.20	2.58
	*β*-cadinene	25.04	1.33
	Dihydroagarofuran	26.43	1.53
	*β*-dihydroagarofuran	26.56	1.05
	*α*-zingiberene	27.06	2.21
	Cedrene	28.72	4.91
	*α*-curcumen	28.89	2.44
	Nerolidol	35.64	1.57
	Elemol	39.25	9.62
	*β*-eudesmol	39.75	1.13
	*γ*-eudesmol	40.50	2.49
	Guaiol	40.94	19.40
	10-epi-*γ*-eudesmol	41.75	14.80
	Valerianol	43.16	23.20
	Drim-7-en-11-ol	50.65	1.84
*Boswelia carterii* Birdw.	Sabinene	4.33	5.51
	3-Carene	5.05	1.39
	*α*-phellandrene	5.54	2.81
	*β*-myrcene	5.65	3.91
	D-limonene	6.68	26.40
	4-Thujanol	6.70	1.49
	prehnitene	9.25	8.65
	Copaene	17.96	1.59
	Bornyl acetate	21.6	1.00
	2-methylene-4,8,8-trimethyl-4-vinyl-bicyclo[5.2.0]nonane	21.93	7.83
	Farnesol	24.6	2.24
	trans-verbenol	25.65	1.55
	*β*-selinene	26.39	2.18
	*γ*-selinene	26.63	1.24
	*γ*-cadinene	28.02	1.67
	*δ*-cadinene	28.12	2.05
	Carveol	31.32	1.03
	Caryophyllene oxide	35.28	2.41
	tau-cadinol	41.74	2.13
*Canarium luzonicum* (Blume) A. Gray	*β*-Phellandrene	4.34	4.54
	α-Phellandrene	5.54	12.20
	D-limonene	6.68	36.40
	cis-Sabinene	6.90	3.06
	o-cymene	9.25	3.35
	*α*-terpinolen	9.75	1.59
	Terpinen-4-ol	22.71	1.15
	*α*-terpineol	26.41	3.83
	Elemol	39.25	16.70
	10-epi-*γ*-eudesmol	41.75	1.59
	Rosifoliol	43.27	1.08
	Elemicin	43.73	9.59
*Cinnamomum camphora* (L.) J. Presl.	Sabinene	4.33	6.07
	*β*-pinene	5.64	1.70
	D-limonene	6.68	25.90
	1,8-cineol	7.07	44.90
	*γ*-terpinene	8.35	1.43
	*o*-cymene	9.25	11.70
*Cinnamomum caphora* var. *linaloolifera* Y. Fuita	1,2-epoxylinalool	16.46	1.27
	Linalool	21.32	96.99
*Citrus x aurantium* L.	*β*-myrcene	5.65	2.32
	D-limonene	6.68	1.57
	1,8-cineole	7.07	2.70
	*β*-ocimene	8.75	2.39
	Linalyl acetate	21.14	63.4
	Caryophyllene	21.92	1.34
	*α*-terpineol	26.41	8.84
	Neryl acetate	27.51	3.77
	Geranyl acetate	28.64	6.02
	Geraniol	30.28	5.31
*Gaultheria procumbens* L.	Methyl salicylate	28.68	98.00
*Litsea cubeba* (Lour.) Pers.	Sabinene	4.33	2.20
	D-limonene	6.68	14.00
	1,8-cineole	7.07	1.62
	6-methyl-5-hepten-2-one	12.11	1.35
	Citronellal	17.86	1.00
	Linalool	21.32	1.82
	Caryophyllene	21.92	1.85
	(Z)-citral	25.56	31.00
	*α*-terpineol	26.41	1.06
	(E)-citral	27.51	35.2
	trans-geraniol	32.01	1.35
	nerolic acid	45.74	1.13
*Melaleuca leucadendron* L.	D-limonene	6.68	8.12
	1,8-Cineole	7.07	49.20
	*γ*-terpinen	8.35	2.91
	o-cymene	9.25	3.16
	*α*-terpinolen	9.75	1.24
	Linalyl acetate	21.14	1.13
	Caryophyllene	21.92	5.65
	2,4-dihydroxy-2-methylpentane	24.12	4.11
	Z,Z,Z-1,5,9,9-tetramethyl-1,4,7,-cycloundecatriene	24.61	2.91
	*α*-terpineol acetate	26.07	1.84
	*α*-terpineol	26.41	9.92
	*α*-selinene	26.64	2.09
	Globulol	38.72	1.90
*Melaleuca ericifolia* Smith.	D-limonene	6.68	2.97
	1,8-cineol	7.07	23.10
	*γ*-terpinene	8.35	2.63
	o-cymene	9.25	3.64
	1,2-epoxylinalool	16.46	2.03
	Ethyl 2-(5-methyl-5-vinyltetrahydrofuran-2-yl)propan-2-yl carbonate	17.61	1.54
	*β*-linalool	21.24	36.70
	2-methylene-4,8,8-trimethyl-4-vinyl-bicyclo[5.2.0]nonane	21.93	1.17
	Alloaromadendrene	22.40	4.73
	Terpinen-4-ol	22.71	2.62
	Aromadendrene	23.75	1.86
	Leden	25.73	1.07
	*α*-terpineol	26.41	4.98
*Pogostemon cabli* (Blanco) Benth.	Patchoulene	17.51	3.28
	*α*-guaien	22.10	18.10
	*α*-patchoulene	23.23	6.09
	1,1,4a-Trimethyl-5,6-dimethylenedecahydronaphthalene	23.33	7.88
	*γ*-patchoulene	23.88	1.10
	Aciphyllene	26.11	3.53
	*γ*-guajene	26.69	18.20
	Patchouli alcohol	41.48	27.30
	Pogostol	42.74	2.65
	Dhelqangin	51.98	2.34
*Citrus limon* (L.) Osbeck	(E)-citral	27.51	2.44
	Sabinene	4.33	3.45
	*β*-myrcene	5.65	2.42
	D-limonene	6.68	67.10
	*α*-terpinolen	9.75	14.20
	*α*-bergamotene	21.89	1.33
	Neral	25.45	1.50
	*β*-bisabolene	27.22	1.30
*Santalum album* L.	*α*-santalene	21.31	5.03
	*α*-bergamotene	21.89	9.68
	α-santalal	41.14	2.54
	*α*-santalol	41.69	59.00
	*β*-santalol	50.36	9.02
	Lanceol	51.12	1.93
	(E)-nuciferol	51.87	1.74
	7-(5-hexynyl)-tricyclo[4.2.2.0(2,5)]dec-7-ene	52.26	1.38
*Vetiveria zizanoides* (L.) Roberty	3,3,5,6,8,8-Hexamethyltricyclo[5.1.0.0(2,4)]oct-5-ene	18.88	1.02
	Tricyclo[6.3.0.0(1,5)]undec-2-en-4-one, 5,9-dimethyl	20.76	2.00
	1,2,4,5-tetraethylbenzene	21.32	4.39
	*α*-muurolene	25.59	1.89
	*α*-cadinene	25.78	1.74
	Selina-5,11-diene	26.16	1.50
	*δ*-cadinene	26.64	1.57
	*α*-vetispirene	27.16	1.77
	*β*-vetispirene	27.42	2.03
	*γ*-vetivenene	30.19	3.30
	*β*-vetivenene	31.27	7.42
	Valencen	32.39	2.19
	3,5,11-Eudesmatriene	34.91	1.65
	7,7-dichlorobicyclo[3.2.0]hept-2-en-6-one	41.06	1.22
	*γ*-himachalene	41.31	2.63
	Ziza-6(13)-en-12-al	41.99	1.02
	Khusiol	45.27	1.12
	*β*-guaiene	45.58	4.43
	Cyclocopacamphenol	46.03	1.66
	Zizanol	47.64	1.03
	(E)-isovalencenal	47.76	1.37
	Vetiselinenol	48.57	1.41
	Vetiverol	50.15	2.22
	Khusenol	50.68	5.24
	Vetiverone	51.09	3.02
	*β*-costol	52.11	3.52
	Khusenic acid	63.32	4.17

Note: * listed are the components that represented min. 1%. Values represent means of three replicate determinations (maximum relative standard deviation ± 5%).

**Table 2:** Antimicrobial resistance of *Staphylococcus* spp.

Name of Bacteria/AMB	C	TET	TIG	TOB
*Stapyloccocus aureus* 18.3	S	S	S	S
*Staphyloccous capitis* 3.6	R	S	S	I
*Staphylococcus epidermidis* 2.2	R	S	S	R
*Staphylococcus epidermidis* 2.4	S	S	S	R
*Staphylococcus epidermidis* 16.2	S	S	S	R
*Staphylococcus epidermidis* 18.4	R	S	S	R
*Staphylococcus epidermidis* 19.5	S	S	S	R
*Staphylococcus epidermidis* 24.2	R	S	S	I
*Staphylococcus epidermidis* 27.3	R	S	S	R
*Staphylococcus haemoliticus* 2.3	R	S	S	R
*Staphylococcus haemoliticus* 14.5	R	S	S	R
*Staphylococcus haemoliticus* 14.6	R	S	S	R
*Staphylococcus haemoliticus* 14.7	S	S	S	R
*Staphylococcus haemoliticus* 14.8	R	S	S	R
*Staphylococcus haemoliticus* 17.1	R	S	S	R
*Staphylococcus haemoliticus* 17.2	R	S	S	R
*Staphylococcus haemoliticus* 17.3	R	S	S	R
*Staphylococcus haemoliticus* 17.5	S	S	S	R
*Staphylococcus haemoliticus* 17.6	R	S	S	S
*Staphylococcus haemoliticus* 19.7	R	S	S	R
*Staphylococcus haemoliticus* 19.8	S	S	S	R
*Staphylococcus haemoliticus* 20.2	S	S	S	R
*Staphylococcus haemoliticus* 20.4	R	S	S	I
*Staphylococcus haemoliticus* 20.5	R	S	S	R
*Staphylococcus haemoliticus* 21.5	R	S	S	S
*Staphylococcus haemoliticus* 21.6	S	S	S	I
*Staphylococcus haemoliticus* 21.7	R	S	S	R
*Staphylococcus haemoliticus* 22.5	R	S	S	R
*Staphylococcus haemoliticus* 24.1	R	S	S	S
*Staphylococcus haemoliticus* 24.2	R	S	S	R
*Staphylococcus haemoliticus* 24.5	R	S	S	S
*Staphylococcus haemoliticus* 24.6	S	S	S	I
*Staphylococcus haemoliticus* 24.7	R	S	S	R
*Staphylococcus haemoliticus* 24.8	R	S	S	R
*Staphylococcus haemoliticus*	R	S	S	S
*Staphylococus hominis* 3.1	S	S	S	R
*Staphylococus hominis* 3.2	R	S	S	R
*Staphylococus hominis* 3.3	R	S	S	I
*Staphylococus hominis* 3.4	R	S	S	R
*Staphylococus hominis* 3.5	S	S	S	R
*Staphylococus hominis* 3.7	R	S	S	S
*Staphylococus hominis* 14.4	R	S	S	R
*Staphylococus hominis* 16.1	R	S	S	S
*Staphylococus hominis* 16.4	S	S	S	R
*Staphylococus hominis* 17.4	R	S	S	R
*Staphylococus hominis* 18.1	R	S	S	R
*Staphylococus hominis* 18.8	R	S	S	I
*Staphylococus hominis* 21.1	R	S	S	S
*Staphylococus hominis* 21.2	R	S	S	I
*Staphylococus hominis* 27.4	R	S	S	S

Note: C—chloramphenicol, TET—tetracycline, TIG—tigecycline, TO—tobramycin, R—resistant, S—sensitive, I—intermediate.

**Table 3:** Antimicrobial activity of essential oils (EOs) with disc diffusion method in mm.

Isolate/EOs	1.	2.	3.	4.	5.	6.	7.
*Stapyloccocus aureus* 18.3	16.50 ± 1.32	11.00 ± 1.00	21.33 ± 1.15	7.67 ± 0.58	24.67 ± 0.58	14.67 ± 0.58	7.33 ± 0.58
*Staphyloccous capitis* 3.6	12.33 ± 0.58	8.67 ± 0.58	24.67 ± 0.58	4.67 ± 0.58	14.67 ± 0.58	7.67 ± 0.58	8.33 ± 0.58
*Staphylococcus epidermidis* 2.2	9.33 ± 0.58	12.67 ± 0.58	20.33 ± 0.58	9.00 ± 1.00	8.33 ± 0.58	15.33 ± 0.58	5.67 ± 0.58
*Staphylococcus epidermidis* 2.4	8.67 ± 0.58	11.67 ± 0.58	22.33 ± 0.58	7.67 ± 0.58	7.33 ± 0.58	15.00 ± 0.00	4.67 ± 0.58
*Staphylococcus epidermidis* 16.2	11.67 ± 0.58	12.67 ± 0.58	21.67 ± 0.58	4.67 ± 0.58	7.67 ± 0.58	16.33 ± 0.58	5.67 ± 0.58
*Staphylococcus epidermidis* 18.4	10.67 ± 0.58	13.33 ± 1.15	19.67 ± 0.58	5.33 ± 0.58	8.33 ± 0.58	15.67 ± 1.15	5.50 ± 0.50
*Staphylococcus epidermidis* 19.5	14.67 ± 0.58	12.67 ± 0.58	20.33 ± 0.58	4.67 ± 0.58	6.33 ± 1.15	16.67 ± 0.58	5.00 ± 1.00
*Staphylococcus epidermidis* 24.2	11.33 ± 1.15	11.67 ± 1.53	19.67 ± 0.58	6.33 ± 0.58	7.33 ± 1.15	16.00 ± 1.00	4.33 ± 0.58
*Staphylococcus epidermidis* 27.3	11.67 ± 1.53	11.67 ± 1.15	20.33 ± 1.15	5.67 ± 0.58	6.67 ± 0.58	17.33 ± 0.58	4.67 ± 0.58
*Staphylococcus haemoliticus* 2.3	8.67 ± 0.58	8.33 ± 0.58	20.67 ± 1.15	4.67 ± 0.58	4.67 ± 0.58	10.33 ± 0.58	5.67 ± 0.58
*Staphylococcus haemoliticus* 14.5	7.67 ± 0.58	8.67 ± 1.15	18.33 ± 0.58	6.33 ± 0.58	5.67 ± 0.58	8.67 ± 0.58	5.33 ± 1.15
*Staphylococcus haemoliticus* 14.6	11.00 ± 1.00	8.67 ± 1.15	19.67 ± 0.58	7.67 ± 0.58	4.67 ± 0.58	8.33 ± 0.58	6.33 ± 1.15
*Staphylococcus haemoliticus* 14.7	7.00 ± 1.00	7.67 ± 0.58	20.33 ± 0.58	8.33 ± 0.58	4.67 ± 1.15	7.67 ± 1.15	4.67 ± 0.58
*Staphylococcus haemoliticus* 14.8	11.00 ± 1.00	8.67 ± 0.58	18.67 ± 0.58	9.33 ± 1.15	5.33 ± 0.58	8.33 ± 0.58	6.00 ± 1.00
*Staphylococcus haemoliticus* 17.1	10.67 ± 1.15	10.67 ± 1.15	18.33 ± 0.58	6.33 ± 1.15	5.67 ± 0.58	6.67 ± 0.58	4.67 ± 0.58
*Staphylococcus haemoliticus* 17.2	9.67 ± 1.15	10.67 ± 0.58	17.33 ± 0.58	5.33 ± 0.58	6.67 ± 0.58	7.33 ± 0.58	5.67 ± 1.15
*Staphylococcus haemoliticus* 17.3	9.00 ± 1.00	8.67 ± 0.58	18.33 ± 0.58	5.33 ± 0.58	6.33 ± 1.15	8.33 ± 0.58	5.33 ± 0.58
*Staphylococcus haemoliticus* 17.5	13.33 ± 1.15	8.67 ± 0.58	17.67 ± 1.15	6.33 ± 0.58	7.33 ± 0.58	6.33 ± 1.15	5.67 ± 0.58
*Staphylococcus haemoliticus* 17.6	11.33 ± 1.15	9.33 ± 0.58	20.33 ± 0.58	5.67 ± 1.15	6.33 ± 0.58	5.67 ± 0.58	5.00 ± 1.00
*Staphylococcus haemoliticus* 19.7	14.33 ± 0.58	7.67 ± 0.58	18.33 ± 1.15	6.00 ± 1.73	7.67 ± 0.58	7.33 ± 0.58	4.67 ± 0.58
*Staphylococcus haemoliticus* 19.8	13.67 ± 1.53	10.67 ± 0.58	18.67 ± 1.15	5.67 ± 1.15	7.33 ± 0.58	8.67 ± 0.58	6.33 ± 0.58
*Staphylococcus haemoliticus* 20.2	9.00 ± 1.00	10.67 ± 1.15	20.33 ± 1.15	5.67 ± 0.58	5.67 ± 0.58	7.33 ± 0.58	4.67 ± 1.15
*Staphylococcus haemoliticus* 20.4	10.33 ± 0.58	8.67 ± 0.58	19.33 ± 1.15	5.00 ± 1.00	6.33 ± 0.58	7.67 ± 1.15	5.67 ± 1.15
*Staphylococcus haemoliticus* 20.5	13.00 ± 1.00	9.00 ± 1.00	18.33 ± 1.15	7.33 ± 0.58	5.33 ± 0.58	7.67 ± 0.58	6.00 ± 1.00
*Staphylococcus haemoliticus* 21.5	14.33 ± 0.58	8.33 ± 0.58	20.33 ± 0.58	7.67 ± 0.58	6.00 ± 1.00	8.33 ± 0.58	5.83 ± 0.29
*Staphylococcus haemoliticus* 21.6	9.33 ± 0.58	9.67 ± 1.15	18.67 ± 0.58	7.33 ± 0.58	6.67 ± 1.15	7.67 ± 0.58	6.17 ± 0.29
*Staphylococcus haemoliticus* 21.7	8.33 ± 0.58	10.33 ± 0.58	17.67 ± 0.58	6.67 ± 0.58	4.67 ± 1.15	6.33 ± 0.58	5.17 ± 0.29
*Staphylococcus haemoliticus* 22.5	7.33 ± 0.58	8.33 ± 0.58	17.33 ± 1.15	5.67 ± 0.58	4.67 ± 0.58	6.33 ± 1.15	4.33 ± 0.58
*Staphylococcus haemoliticus* 24.1	11.67 ± 0.58	10.33 ± 0.58	18.67 ± 0.58	6.33 ± 1.15	5.33 ± 1.53	6.67 ± 1.15	5.67 ± 1.15
*Staphylococcus haemoliticus* 24.2	10.33 ± 0.58	10.67 ± 0.58	19.33 ± 1.15	8.67 ± 1.15	5.67 ± 1.15	6.67 ± 0.58	4.67 ± 1.15
*Staphylococcus haemoliticus* 24.5	12.17 ± 0.29	9.67 ± 0.58	18.67 ± 0.58	7.67 ± 0.58	6.67 ± 0.58	6.00 ± 1.00	5.33 ± 0.58
*Staphylococcus haemoliticus* 24.6	10.67 ± 0.58	8.33 ± 0.58	17.67 ± 0.58	8.33 ± 1.15	5.67 ± 0.58	5.67 ± 0.58	4.33 ± 0.58
*Staphylococcus haemoliticus* 24.7	12.00 ± 2.00	8.33 ± 0.58	19.33 ± 1.15	8.00 ± 1.00	6.33 ± 1.15	4.67 ± 0.58	4.67 ± 0.58
*Staphylococcus haemoliticus* 24.8	10.33 ± 0.58	8.33 ± 0.58	18.33 ± 0.58	7.67 ± 0.58	6.33 ± 0.58	5.33 ± 0.58	5.83 ± 0.76
*Staphylococcus haemoliticus*	11.33 ± 0.58	12.67 ± 0.58	20.33 ± 0.58	8.33 ± 0.58	7.67 ± 0.58	6.33 ± 0.58	4.67 ± 0.58
*Staphylococus hominis* 3.1	11.33 ± 0.58	10.33 ± 0.58	21.67 ± 0.58	10.67 ± 0.58	18.33 ± 0.58	8.00 ± 1.73	3.33 ± 1.15
*Staphylococus hominis* 3.2	9.33 ± 0.58	11.33 ± 1.15	22.00 ± 1.73	9.33 ± 0.58	19.67 ± 0.58	8.33 ± 0.58	3.67 ± 0.58
*Staphylococus hominis* 3.3	7.67 ± 0.58	10.00 ± 1.73	21.33 ± 0.58	8.33 ± 0.58	19.33 ± 0.58	8.00 ± 1.73	4.33 ± 0.58
*Staphylococus hominis* 3.4	11.67 ± 0.58	8.33 ± 0.58	21.33 ± 0.58	9.00 ± 1.00	18.33 ± 0.58	7.33 ± 1.15	4.67 ± 0.58
*Staphylococus hominis* 3.5	9.67 ± 0.58	9.67 ± 0.58	21.67 ± 0.58	8.33 ± 0.58	20.33 ± 0.58	8.67 ± 0.58	4.33 ± 0.58
*Staphylococus hominis* 3.7	10.33 ± 1.15	10.33 ± 1.15	18.67 ± 0.58	8.67 ± 0.58	21.67 ± 0.58	8.00 ± 1.00	4.33 ± 0.58
*Staphylococus hominis* 14.4	10.67 ± 1.15	9.33 ± 1.15	20.33 ± 0.58	7.33 ± 0.58	18.67 ± 1.15	7.33 ± 1.15	3.67 ± 0.58
*Staphylococus hominis* 16.1	10.67 ± 1.53	8.67 ± 0.58	18.67 ± 0.58	7.67 ± 1.15	17.67 ± 1.15	7.33 ± 0.58	3.67 ± 1.15
*Staphylococus hominis* 16.4	9.33 ± 0.58	8.33 ± 0.58	20.33 ± 0.58	7.33 ± 1.15	19.67 ± 0.58	9.33 ± 0.58	3.67 ± 0.58
*Staphylococus hominis* 17.4	11.33 ± 1.15	9.00 ± 1.00	21.67 ± 0.58	8.33 ± 0.58	19.33 ± 0.58	7.67 ± 0.58	5.33 ± 0.58
*Staphylococus hominis* 18.1	12.67 ± 1.15	10.67 ± 0.58	20.67 ± 0.58	8.67 ± 0.58	18.33 ± 0.58	8.67 ± 0.58	5.67 ± 0.58
*Staphylococus hominis* 18.8	9.00 ± 1.00	11.67 ± 0.58	21.67 ± 0.58	8.67 ± 1.15	17.33 ± 0.58	7.67 ± 0.58	4.33 ± 0.58
*Staphylococus hominis* 21.1	11.33 ± 0.58	11.67 ± 0.58	20.50 ± 0.50	8.55 ± 0.50	18.33 ± 0.58	8.33 ± 0.58	4.67 ± 0.58
*Staphylococus hominis* 21.2	11.00 ± 1.00	11.00 ± 1.00	21.33 ± 0.58	7.67 ± 0.58	19.33 ± 1.15	8.67 ± 1.15	4.00 ± 0.00
*Staphylococus hominis* 27.4	10.67 ± 1.15	9.33 ± 0.58	22.33 ± 0.58	7.67 ± 0.58	19.33 ± 1.53	7.67 ± 0.58	4.33 ± 1.15

Note: 1—Amyris balsamifera L., 2—Boswelia carterii Birdw., 3—Canarium luzonicum (Blume) A. Gray, 4—Cinnamomum camphora (L.) J. Presl., 5—Cinnamomum camphora var. linaloolifera Y. Fuita, 6—Citrus x aurantium L., 7—Gaultheria procumbens L.

**Table 4:** Antimicrobial activity of EOs with disc diffusion method in mm.

Isolate/EOs	8.	9.	10.	11.	12.	13.	14.
*Stapyloccocus aureus* 18.3	23.33 ± 0.58	4.67 ± 0.58	14.67 ± 0.58	9.00 ± 1.00	10.67 ± 1.15	5.33 ± 0.58	11.33 ± 0.58
*Staphyloccous capitis* 3.6	25.33 ± 0.58	3.67 ± 1.15	8.67 ± 0.58	8.33 ± 0.58	12.67 ± 1.15	7.33 ± 0.58	12.67 ± 0.58
*Staphylococcus epidermidis* 2.2	14.66 ± 0.58	4.33 ± 0.58	6.67 ± 0.58	7.33 ± 0.58	8.33 ± 0.58	4.33 ± 0.58	6.67 ± 1.15
*Staphylococcus epidermidis* 2.4	12.67 ± 0.58	4.00 ± 1.00	5.67 ± 0.58	6.33 ± 0.58	8.67 ± 1.15	4.33 ± 0.58	6.67 ± 1.15
*Staphylococcus epidermidis* 16.2	14.33 ± 0.58	3.67 ± 0.58	6.33 ± 1.15	6.00 ± 1.00	9.00 ± 1.00	5.00 ± 1.00	6.67 ± 0.58
*Staphylococcus epidermidis* 18.4	14.50 ± 0.87	4.67 ± 0.58	7.33 ± 0.58	5.67 ± 1.15	9.67 ± 1.15	5.33 ± 0.58	7.00 ± 1.73
*Staphylococcus epidermidis* 19.5	11.67 ± 0.58	3.67 ± 0.58	6.33 ± 0.58	5.67 ± 0.58	8.33 ± 0.58	4.33 ± 1.15	7.67 ± 0.58
*Staphylococcus epidermidis* 24.2	12.67 ± 1.53	3.33 ± 0.58	7.33 ± 0.58	6.67 ± 0.58	9.33 ± 0.58	5.33 ± 0.58	8.33 ± 0.58
*Staphylococcus epidermidis* 27.3	11.33 ± 1.15	4.67 ± 0.58	5.67 ± 0.58	5.33 ± 0.58	8.67 ± 0.58	4.67 ± 0.58	8.33 ± 0.58
*Staphylococcus haemoliticus* 2.3	12.33 ± 0.58	4.67 ± 1.15	5.33 ± 0.58	10.67 ± 1.15	10.33 ± 0.58	5.33 ± 0.58	8.67 ± 0.58
*Staphylococcus haemoliticus* 14.5	9.33 ± 0.58	3.67 ± 1.15	5.33 ± 0.58	10.33 ± 0.58	9.00 ± 1.00	4.00 ± 1.00	7.33 ± 0.58
*Staphylococcus haemoliticus* 14.6	9.33 ± 1.15	3.33 ± 0.58	6.33 ± 0.58	11.00 ± 1.00	8.33 ± 0.58	4.67 ± 0.58	7.00 ± 1.73
*Staphylococcus haemoliticus* 14.7	9.67 ± 1.15	4.67 ± 1.15	5.67 ± 1.15	11.33 ± 1.15	9.33 ± 1.15	5.00 ± 1.00	7.33 ± 0.58
*Staphylococcus haemoliticus* 14.8	9.33 ± 1.53	4.67 ± 0.58	5.67 ± 0.58	10.33 ± 2.08	9.67 ± 1.15	4.67 ± 0.58	6.33 ± 0.58
*Staphylococcus haemoliticus* 17.1	10.67 ± 1.15	3.67 ± 0.58	5.67 ± 1.53	12.33 ± 0.58	8.33 ± 1.53	4.67 ± 1.15	7.67 ± 1.53
*Staphylococcus haemoliticus* 17.2	10.00 ± 1.73	3.67 ± 0.58	5.33 ± 0.58	10.67 ± 0.58	10.00 ± 2.00	5.00 ± 0.00	6.33 ± 0.58
*Staphylococcus haemoliticus* 17.3	10.33 ± 1.53	4.33 ± 0.58	5.33 ± 1.15	11.67 ± 0.58	9.67 ± 1.53	5.50 ± 0.50	8.33 ± 0.58
*Staphylococcus haemoliticus* 17.5	8.00 ± 1.00	4.67 ± 0.58	6.33 ± 0.58	12.67 ± 1.15	8.33 ± 0.58	4.67 ± 0.58	8.33 ± 1.53
*Staphylococcus haemoliticus* 17.6	6.67 ± 1.53	4.33 ± 0.58	5.67 ± 0.58	11.33 ± 1.15	9.00 ± 1.00	5.17 ± 0.76	8.67 ± 0.58
*Staphylococcus haemoliticus* 19.7	6.33 ± 1.15	3.33 ± 1.15	6.33 ± 0.58	9.33 ± 1.15	7.67 ± 0.58	5.67 ± 1.15	7.67 ± 0.58
*Staphylococcus haemoliticus* 19.8	7.33 ± 0.58	4.33 ± 1.15	5.67 ± 0.58	10.67 ± 0.58	8.33 ± 0.58	5.33 ± 0.58	6.67 ± 1.15
*Staphylococcus haemoliticus* 20.2	6.67 ± 1.15	3.67 ± 1.15	6.33 ± 1.15	10.33 ± 2.08	9.33 ± 1.15	3.67 ± 0.58	6.67 ± 0.58
*Staphylococcus haemoliticus* 20.4	8.33 ± 1.15	4.00 ± 0.00	6.00 ± 1.73	10.67 ± 1.15	10.67 ± 1.53	6.00 ± 1.00	5.33 ± 0.58
*Staphylococcus haemoliticus* 20.5	8.67 ± 0.58	3.67 ± 1.15	5.33 ± 0.58	12.33 ± 0.58	10.67 ± 0.58	4.67 ± 0.58	5.67 ± 0.58
*Staphylococcus haemoliticus* 21.5	8.33 ± 0.58	3.83 ± 0.76	6.67 ± 1.53	11.67 ± 0.58	10.67 ± 1.15	5.33 ± 1.15	4.66 ± 1.15
*Staphylococcus haemoliticus* 21.6	9.33 ± 0.58	4.33 ± 0.58	8.67 ± 0.58	11.33 ± 1.15	9.00 ± 1.00	4.67 ± 0.58	6.33 ± 1.15
*Staphylococcus haemoliticus* 21.7	8.00 ± 1.73	4.33 ± 0.58	8.67 ± 1.15	11.33 ± 0.58	10.67 ± 1.15	5.33 ± 0.58	6.67 ± 1.15
*Staphylococcus haemoliticus* 22.5	7.33 ± 0.58	3.67 ± 0.58	8.33 ± 1.15	10.67 ± 1.15	9.00 ± 1.00	5.00 ± 1.73	6.33 ± 0.58
*Staphylococcus haemoliticus* 24.1	10.00 ± 1.00	4.33 ± 0.58	8.00 ± 1.00	10.67 ± 0.58	9.00 ± 1.73	5.00 ± 0.87	7.00 ± 1.73
*Staphylococcus haemoliticus* 24.2	10.33 ± 1.53	4.67 ± 0.58	8.67 ± 1.15	10.67 ± 1.15	9.00 ± 1.00	5.33 ± 0.58	6.33 ± 0.58
*Staphylococcus haemoliticus* 24.5	9.67 ± 1.15	4.67 ± 1.15	8.33 ± 1.15	12.67 ± 1.15	10.67 ± 1.15	5.67 ± 1.15	7.33 ± 0.58
*Staphylococcus haemoliticus* 24.6	7.67 ± 0.58	4.67 ± 0.58	9.67 ± 0.58	10.67 ± 0.58	9.33 ± 1.15	5.33 ± 0.58	6.67 ± 1.15
*Staphylococcus haemoliticus* 24.7	8.00 ± 1.00	5.67 ± 0.58	8.33 ± 0.58	12.33 ± 0.58	9.33 ± 1.53	4.33 ± 0.58	6.67 ± 1.53
*Staphylococcus haemoliticus* 24.8	8.33 ± 0.58	4.33 ± 0.58	9.33 ± 0.58	10.67 ± 0.58	8.67 ± 1.15	4.67 ± 0.58	5.67 ± 0.58
*Staphylococcus haemoliticus*	6.67 ± 0.58	4.67 ± 0.58	8.67 ± 1.15	12.67 ± 1.15	9.67 ± 0.58	5.33 ± 0.58	5.33 ± 0.58
*Staphylococus hominis* 3.1	21.33 ± 1.15	6.67 ± 0.58	15.33 ± 0.58	8.67 ± 1.15	8.33 ± 0.58	7.67 ± 0.58	10.67 ± 1.15
*Staphylococus hominis* 3.2	20.67 ± 0.58	5.67 ± 0.58	14.33 ± 0.58	8.33 ± 0.58	7.33 ± 0.58	6.67 ± 0.58	11.33 ± 0.58
*Staphylococus hominis* 3.3	19.33 ± 0.58	6.33 ± 1.15	15.33 ± 0.58	10.33 ± 0.58	8.33 ± 0.58	7.67 ± 0.58	11.67 ± 0.58
*Staphylococus hominis* 3.4	21.00 ± 1.73	6.67 ± 1.15	14.33 ± 0.58	10.67 ± 1.15	7.67 ± 0.58	8.67 ± 0.58	11.67 ± 1.53
*Staphylococus hominis* 3.5	21.67 ± 1.15	5.67 ± 0.58	13.33 ± 0.58	9.67 ± 0.58	7.67 ± 1.15	7.67 ± 0.58	11.67 ± 0.58
*Staphylococus hominis* 3.7	20.67 ± 1.15	5.33 ± 1.53	14.33 ± 0.58	12.33 ± 0.58	8.33 ± 0.58	7.67 ± 0.58	12.33 ± 0.58
*Staphylococus hominis* 14.4	21.67 ± 0.58	4.67 ± 0.58	13.67 ± 1.53	10.33 ± 0.58	7.67 ± 0.58	8.33 ± 0.58	12.67 ± 1.15
*Staphylococus hominis* 16.1	22.67 ± 1.15	5.67 ± 0.58	11.33 ± 1.15	12.67 ± 1.15	6.67 ± 1.15	6.67 ± 1.15	11.33 ± 1.15
*Staphylococus hominis* 16.4	20.33 ± 1.15	6.67 ± 0.58	14.33 ± 0.58	10.33 ± 1.15	6.33 ± 0.58	7.33 ± 0.58	10.33 ± 0.58
*Staphylococus hominis* 17.4	22.33 ± 0.58	6.33 ± 0.58	12.67 ± 1.15	11.33 ± 1.15	5.33 ± 1.15	8.67 ± 0.58	10.67 ± 0.58
*Staphylococus hominis* 18.1	22.67 ± 0.58	6.00 ± 1.00	11.33 ± 1.15	9.33 ± 0.58	6.33 ± 1.15	7.33 ± 0.58	9.33 ± 0.58
*Staphylococus hominis* 18.8	19.33 ± 0.58	7.00 ± 1.73	11.67 ± 1.15	11.67 ± 1.53	5.67 ± 1.15	8.67 ± 0.58	11.33 ± 0.58
*Staphylococus hominis* 21.1	18.67 ± 0.58	6.33 ± 0.58	12.67 ± 0.50	13.67 ± 1.53	7.33 ± 1.15	6.33 ± 1.53	11.67 ± 0.58
*Staphylococus hominis* 21.2	18.33 ± 0.58	7.67 ± 0.58	12.00 ± 1.73	11.33 ± 1.53	6.33 ± 0.58	7.00 ± 1.73	12.33 ± 0.58
*Staphylococus hominis* 27.4	19.00 ± 1.73	7.67 ± 1.15	11.67 ± 0.58	12.67 ± 0.58	7.33 ± 0.58	8.33 ± 0.58	12.33 ± 0.58

Note: 8—Litsea cubeba (Lour.) Pers., 9—Melaleuca leucadendron L., 10—Melaleuca ericifolia Smith., 11—Pogostemon cabli (Blanco) Benth., 12—Citrus limon (L.) Osbeck, 13—Santalum album L., 14—Vetiveria zizanoides (L.) Roberty.

**Table 5:** Antimicrobial activity of EO detected with minimal inhibitory concentration in µL/mL.

Microorganism/EOs	1.	2.	3.	4.	5.	6.	7.
*Stapyloccocus aureus* 18.3	1.56	1.56	0.39	25.00	0.39	3.12	12.50
*Staphyloccous capitis* 3.6	1.56	3.12	0.78	25.00	1.56	12.50	12.50
*Staphylococcus epidermidis* 2.2	3.12	1.56	0.78	12.50	3.12	3.12	25.00
*Staphylococcus epidermidis* 2.4	3.12	1.56	0.78	25.00	3.12	3.12	25.00
*Staphylococcus epidermidis* 16.2	3.12	1.56	0.39	25.00	3.12	3.12	25.00
*Staphylococcus epidermidis* 18.4	3.12	1.56	1.56	25.00	3.12	3.12	25.00
*Staphylococcus epidermidis* 19.5	1.56	1.56	0.78	25.00	3.12	3.12	25.00
*Staphylococcus epidermidis* 24.2	3.12	1.56	1.56	25.00	3.12	3.12	25.00
*Staphylococcus epidermidis* 27.3	3.12	1.56	0.78	25.00	3.12	3.12	25.00
*Staphylococcus haemoliticus* 2.3	6.25	6.25	0.78	25.00	12.50	6.25	25.00
*Staphylococcus haemoliticus* 14.5	6.25	6.25	1.56	25.00	12.50	12.50	25.00
*Staphylococcus haemoliticus* 14.6	3.12	6.25	1.56	25.00	12.50	12.50	25.00
*Staphylococcus haemoliticus* 14.7	12.50	6.25	0.78	3.12	12.50	12.50	25.00
*Staphylococcus haemoliticus* 14.8	3.12	6.25	1.56	3.12	12.50	12.50	25.00
*Staphylococcus haemoliticus* 17.1	3.12	3.12	1.56	25.00	12.50	12.50	25.00
*Staphylococcus haemoliticus* 17.2	3.12	3.12	1.56	25.00	12.50	12.50	25.00
*Staphylococcus haemoliticus* 17.3	3.12	6.25	1.56	25.00	12.50	12.50	25.00
*Staphylococcus haemoliticus* 17.5	1.56	6.25	1.56	25.00	6.25	25.00	25.00
*Staphylococcus haemoliticus* 17.6	3.12	6.25	0.78	25.00	6.25	25.00	25.00
*Staphylococcus haemoliticus* 19.7	1.56	6.25	1.56	25.00	6.25	25.00	25.00
*Staphylococcus haemoliticus* 19.8	1.56	3.12	1.56	25.00	6.25	12.50	12.50
*Staphylococcus haemoliticus* 20.2	6.25	3.12	0.78	25.00	12.50	12.50	25.00
*Staphylococcus haemoliticus* 20.4	6.25	6.25	1.56	25.00	12.50	12.50	25.00
*Staphylococcus haemoliticus* 20.5	1.56	6.25	1.56	25.00	25.00	12.50	12.50
*Staphylococcus haemoliticus* 21.5	1.56	6.25	0.78	25.00	12.50	12.50	25.00
*Staphylococcus haemoliticus* 21.6	3.12	6.25	1.56	25.00	12.50	12.50	12.50
*Staphylococcus haemoliticus* 21.7	6.25	3.12	1.56	25.00	25.00	12.50	25.00
*Staphylococcus haemoliticus* 22.5	6.25	6.25	1.56	25.00	25.00	12.50	25.00
*Staphylococcus haemoliticus* 24.1	3.12	1.56	1.56	25.00	12.50	12.50	25.00
*Staphylococcus haemoliticus* 24.2	3.12	1.56	1.56	25.00	12.50	12.50	25.00
*Staphylococcus haemoliticus* 24.5	1.56	3.12	1.56	25.00	12.50	12.50	25.00
*Staphylococcus haemoliticus* 24.6	1.56	3.12	1.56	25.00	12.50	25.00	25.00
*Staphylococcus haemoliticus* 24.7	1.56	3.12	1.56	25.00	12.50	25.00	25.00
*Staphylococcus haemoliticus* 24.8	1.56	3.12	1.56	25.00	12.50	25.00	25.00
*Staphylococcus haemoliticus*	1.56	1.56	0.78	25.00	12.50	12.50	25.00
*Staphylococus hominis* 3.1	1.56	1.56	0.78	12.50	3.12	12.50	25.00
*Staphylococus hominis* 3.2	3.12	1.56	0.78	12.50	3.12	12.50	25.00
*Staphylococus hominis* 3.3	6.25	1.56	0.78	12.50	3.12	12.50	25.00
*Staphylococus hominis* 3.4	3.12	3.12	0.78	12.50	3.12	12.50	25.00
*Staphylococus hominis* 3.5	3.12	3.12	0.78	12.50	1.56	12.50	25.00
*Staphylococus hominis* 3.7	3.12	1.56	1.56	12.50	0.78	12.50	25.00
*Staphylococus hominis* 14.4	3.12	3.12	0.78	12.50	1.56	12.50	25.00
*Staphylococus hominis* 16.1	3.12	3.12	1.56	6.25	1.56	12.50	25.00
*Staphylococus hominis* 16.4	3.12	3.12	0.78	12.50	3.12	12.50	25.00
*Staphylococus hominis* 17.4	1.56	3.12	0.78	25.00	3.12	12.50	25.00
*Staphylococus hominis* 18.1	1.56	3.12	0.78	25.00	3.12	12.50	25.00
*Staphylococus hominis* 18.8	3.12	3.12	0.78	12.50	1.56	12.50	25.00
*Staphylococus hominis* 21.1	3.12	3.12	0.78	12.50	1.56	12.50	25.00
*Staphylococus hominis* 21.2	3.12	3.12	0.78	25.00	1.56	12.50	25.00
*Staphylococus hominis* 27.4	3.12	6.25	0.39	25.00	3.12	12.50	25.00

Note: 1—Amyris balsamifera L., 2—Boswelia carterii Birdw., 3—Canarium luzonicum (Blume) A. Gray, 4—Cinnamomum camphora (L.) J. Presl., 5—Cinnamomum camphora var. linaloolifera Y. Fuita, 6—Citrus x aurantium L., 7—Gaultheria procumbens L.

**Table 6:** Minimal inhibitory concentration of EOs in µL/mL.

Name of bacteria/EOs	8.	9.	10.	11.	12.	13.	14.
*Stapyloccocus aureus* 18.3	0.39	25.00	3.12	3.12	3.12	12.50	3.12
*Staphyloccous capitis* 3.6	0.39	25.00	6.25	3.12	3.12	6.25	3.12
*Staphylococcus epidermidis* 2.2	1.56	25.00	12.50	6.25	6.25	12.50	25.00
*Staphylococcus epidermidis* 2.4	1.56	25.00	12.50	6.25	6.25	12.50	25.00
*Staphylococcus epidermidis* 16.2	1.56	25.00	12.50	6.25	6.25	12.50	25.00
*Staphylococcus epidermidis* 18.4	1.56	25.00	12.50	6.25	6.25	12.50	25.00
*Staphylococcus epidermidis* 19.5	1.56	25.00	12.50	6.25	6.25	12.50	12.50
*Staphylococcus epidermidis* 24.2	1.56	25.00	12.50	6.25	6.25	12.50	12.50
*Staphylococcus epidermidis* 27.3	1.56	25.00	12.50	6.25	6.25	12.50	12.50
*Staphylococcus haemoliticus* 2.3	1.56	25.00	12.50	3.12	3.12	12.50	12.50
*Staphylococcus haemoliticus* 14.5	3.12	25.00	12.50	3.12	3.12	12.50	12.50
*Staphylococcus haemoliticus* 14.6	3.12	25.00	12.50	3.12	3.12	12.50	12.50
*Staphylococcus haemoliticus* 14.7	3.12	25.00	12.50	3.12	3.12	12.50	12.50
*Staphylococcus haemoliticus* 14.8	3.12	25.00	12.50	3.12	3.12	12.50	12.50
*Staphylococcus haemoliticus* 17.1	3.12	25.00	12.50	3.12	3.12	12.50	12.50
*Staphylococcus haemoliticus* 17.2	3.12	25.00	12.50	3.12	3.12	12.50	12.50
*Staphylococcus haemoliticus* 17.3	3.12	25.00	12.50	3.12	3.12	12.50	12.50
*Staphylococcus haemoliticus* 17.5	6.25	25.00	12.50	3.12	3.12	25.00	12.50
*Staphylococcus haemoliticus* 17.6	6.25	25.00	12.50	3.12	3.12	25.00	12.50
*Staphylococcus haemoliticus* 19.7	6.25	25.00	12.50	3.12	6.25	25.00	12.50
*Staphylococcus haemoliticus* 19.8	6.25	25.00	12.50	3.12	6.25	25.00	12.50
*Staphylococcus haemoliticus* 20.2	6.25	25.00	12.50	3.12	6.25	25.00	12.50
*Staphylococcus haemoliticus* 20.4	6.25	25.00	12.50	3.12	3.12	25.00	25.00
*Staphylococcus haemoliticus* 20.5	6.25	25.00	12.50	3.12	3.12	25.00	25.00
*Staphylococcus haemoliticus* 21.5	6.25	25.00	12.50	3.12	3.12	25.00	25.00
*Staphylococcus haemoliticus* 21.6	6.25	25.00	6.25	3.12	3.12	25.00	25.00
*Staphylococcus haemoliticus* 21.7	6.25	25.00	6.25	3.12	3.12	25.00	25.00
*Staphylococcus haemoliticus* 22.5	6.25	25.00	6.25	3.12	3.12	25.00	25.00
*Staphylococcus haemoliticus* 24.1	3.12	25.00	6.25	3.12	3.12	25.00	25.00
*Staphylococcus haemoliticus* 24.2	3.12	25.00	6.25	3.12	3.12	25.00	25.00
*Staphylococcus haemoliticus* 24.5	3.12	25.00	6.25	3.12	3.12	25.00	25.00
*Staphylococcus haemoliticus* 24.6	6.25	25.00	6.25	3.12	3.12	25.00	25.00
*Staphylococcus haemoliticus* 24.7	6.25	25.00	6.25	3.12	3.12	25.00	25.00
*Staphylococcus haemoliticus* 24.8	6.25	25.00	6.25	3.12	3.12	25.00	25.00
*Staphylococcus haemoliticus*	6.25	25.00	6.25	3.12	3.12	25.00	25.00
*Staphylococus hominis* 3.1	0.78	12.50	3.12	6.25	3.12	6.25	3.12
*Staphylococus hominis* 3.2	0.78	12.50	3.12	6.25	6.25	6.25	3.12
*Staphylococus hominis* 3.3	0.78	12.50	3.12	3.12	3.12	6.25	3.12
*Staphylococus hominis* 3.4	0.78	12.50	3.12	3.12	3.12	6.25	3.12
*Staphylococus hominis* 3.5	0.78	25.00	3.12	3.12	3.12	6.25	3.12
*Staphylococus hominis* 3.7	0.78	25.00	3.12	3.12	3.12	6.25	3.12
*Staphylococus hominis* 14.4	0.78	25.00	3.12	3.12	3.12	6.25	3.12
*Staphylococus hominis* 16.1	0.78	25.00	3.12	3.12	3.12	6.25	3.12
*Staphylococus hominis* 16.4	0.78	12.50	3.12	3.12	3.12	6.25	3.12
*Staphylococus hominis* 17.4	0.78	12.50	3.12	3.12	3.12	6.25	3.12
*Staphylococus hominis* 18.1	0.78	12.50	3.12	6.25	3.12	6.25	3.12
*Staphylococus hominis* 18.8	0.78	12.50	3.12	3.12	3.12	6.25	3.12
*Staphylococus hominis* 21.1	0.78	12.50	3.12	3.12	6.25	6.25	3.12
*Staphylococus hominis* 21.2	0.78	12.50	3.12	3.12	6.25	6.25	3.12
*Staphylococus hominis* 27.4	0.78	12.50	3.12	3.12	6.25	6.25	3.12

Note: 8—Litsea cubeba (Lour.) Pers., 9—Melaleuca leucadendron L., 10—Melaleuca ericifolia Smith., 11—Pogostemon cabli (Blanco) Benth., 12—Citrus limon (L.) Osbeck, 13—Santalum album L., 14—Vetiveria zizanoides (L.) Roberty.

**Table 7:** Differences between the analysed essential oils in their activity against *Staphylococcus* spp.

Essential Oil	Average/Standard Deviation/Statistical Difference
*A. balsamifera* L.	10.82 ± 2.09 ^a^
*B. carterii* Birdw.	9.95 ± 1.64 ^b,a^
*C. luzonicum* (Blume) A. Gray	19.84 ± 1.94 ^c,a,b^
*C. camphora* (L.) J. Presl.	7.18 ± 1.60 ^d,a,b,c^
*C. camphora* var. *linaloolifera* Y. Fuita	10.65 ± 6.30 ^e,c,d^
*C. x aurantium* L.	8.84 ± 3.33 ^f,a,b,c,d,e^
*G. procumbens* L.	5.07 ± 1.13 ^g,a,b,c,d,e,f^
*L. cubeba* (Lour.) Pers.	13.54 ± 5.90 ^h,a,b,c,d,e,g^
*M. leucadendron* L.	4.82 ± 1.34 ^i,a,b,c,d,e,f,h^
*M. ericifolia* Smith.	8.93 ± 3.40 ^j,a,b,c,d,e,g,h,i^
*P. cabli* (Blanco) Benth.	10.29 ± 2.20 ^k,c,d,g,h,i,j^
*C. limon* (L.) Osbeck	8.71 ± 1.66 ^l,a,b,c,d,e,g,h,i,k^
*S. album* L.	5.81 ± 1.53 ^m,a,b,c,d,e,g,h,i,j,k,l^
*V. zizanoides* (L.) Roberty	8.47 ± 2.49 ^n,a,b,c,d,e,g,h,i,k,m^

Note: Individual letters (a–n) in upper case indicate the statistical difference. *p* ≤ 0.05.

**Table 8:** List of essential oils.

Botanical Species	Common Name	Family	Part
*Amyris balsamifera* L.	amyris	Rutaceae	crushed wood
*Boswellia carterii* Birdw.	frankincense	Burseraceae	resin
*Canarium luzonicum* (Blume) A. Gray	elemi	Burseraceae	resin
*Cinnamomum camphora* (L.) J. Presl.	camphor three bark	Lauraceae	wood, branches
*Cinnamomum camphora* var. *linaloolifera* Y. Fuita	ho leaf	Lauraceae	leaves
*Citrus x aurantium* L.	petitgrain	Rutaceae	leaves
*Gaultheria procumbens* L.	wintergreen	Ericaceae	leaves
*Litsea cubeba* (Lour.) Pers.	litsea cubeba fruit	Lauraceae	fruits
*Melaleuca leucadendron* L.	cajeput	Myrtaceae	shoots of leaves
*Melaleuca ericifolia* Smith.	rosalina	Myrtaceae	branches
*Pogostemon cabli* (Blanco) Benth.	patchouli	Lamiaceae	fermented leaves
*Citrus limon* (L.) Osbeck	lemon	Rutaceae	fruits
*Santalum album* L.	sandalwood	Santalaceae	crushed wood
*Vetiveria zizanoides* (L.) Roberty	vetiver	Poaceae	dried roots
